# Off-Policy Evaluation of the Performance of a Robot Swarm: Importance Sampling to Assess Potential Modifications to the Finite-State Machine That Controls the Robots

**DOI:** 10.3389/frobt.2021.625125

**Published:** 2021-04-29

**Authors:** Federico Pagnozzi, Mauro Birattari

**Affiliations:** IRIDIA, Université libre de Bruxelles, Brussels, Belgium

**Keywords:** swarm robotics, control software architecture, automatic design, reinforcement learning, importance sampling

## Abstract

Due to the decentralized, loosely coupled nature of a swarm and to the lack of a general design methodology, the development of control software for robot swarms is typically an iterative process. Control software is generally modified and refined repeatedly, either manually or automatically, until satisfactory results are obtained. In this paper, we propose a technique based on off-policy evaluation to estimate how the performance of an instance of control software—implemented as a probabilistic finite-state machine—would be impacted by modifying the structure and the value of the parameters. The proposed technique is particularly appealing when coupled with automatic design methods belonging to the AutoMoDe family, as it can exploit the data generated during the design process. The technique can be used either to reduce the complexity of the control software generated, improving therefore its readability, or to evaluate perturbations of the parameters, which could help in prioritizing the exploration of the neighborhood of the current solution within an iterative improvement algorithm. To evaluate the technique, we apply it to control software generated with an AutoMoDe method, Chocolate−6S . In a first experiment, we use the proposed technique to estimate the impact of removing a state from a probabilistic finite-state machine. In a second experiment, we use it to predict the impact of changing the value of the parameters. The results show that the technique is promising and significantly better than a naive estimation. We discuss the limitations of the current implementation of the technique, and we sketch possible improvements, extensions, and generalizations.

## 1 Introduction

In this paper, we investigate the use of off-policy evaluation to estimate the performance of a swarm of robots. In swarm robotics (Dorigo et al., 2014), a group of robots act in coordination to perform a given mission. This engineering discipline is inspired by the principles of swarm intelligence (Dorigo and Birattari, 2007). The behavior of the swarm is determined by the local interactions of the robots with each other and with the environment. In a robot swarm, there is no single point of failure and additional robots can be added to the swarm without changing the control software. Unfortunately, these same features make designing the control software of the individual robots comprised in a swarm a complex endeavor. In fact, with the exception of some specific cases (Brambilla et al., 2015; Reina et al., 2015; Lopes et al., 2016), a general design methodology has yet to be proposed (Francesca and Birattari, 2016). Typically, the design of the control software of the individual robots comprised in a swarm is an iterative improvement process based on trial and error and heavily relies on the experience and intuition of the designer (Francesca et al., 2014). Automatic design has shown to be a valid alternative to manual design (Francesca and Birattari, 2016; Birattari et al., 2019). Automatic design methods work by formulating the design problem as an optimization problem, which is then solved using generally available heuristic methods. The solution of the optimization problem is an instance of control software and the solution quality is a measure of its performance. In other words, the optimal solution of such optimization problem is the control software that maximizes an appropriate mission-dependent performance metric. Reviews of the swarm robotics literature can be found in Garattoni and Birattari (2016) and Brambilla et al. (2013), while in depth reviews of automatic design in swarm robotics can be found in Francesca and Birattari (2016); Bredeche et al. (2018); Birattari et al. (2020).

In this study, we focus on control software implemented as a probabilistic finite-state machine (PFSM): a graph where each node represents a low-level behavior of the robot and each edge represents a transition from a low-level behavior to another. When the condition associated to a transition is verified, the transition is performed and the current state changes. In a probabilistic finite-state machine, each transition whose associated condition is verified may take place with a certain probability. This control software architecture is human readable and modular—states and transitions can be defined once and easily reused or changed. Due to these characteristics, finite-state machines have been commonly used in manual design as well as in automatic design methods such as the ones belonging to the AutoMoDe family (Francesca et al., 2014).

**FIGURE 1:**
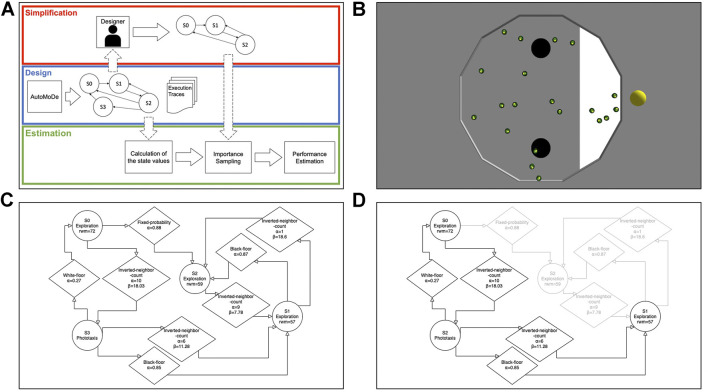
In panel **(A)**, we show the workflow of the experiments. During the design process, AutoMoDe produces several finite-state machines—such as the one composed of four states shown in panel **(C)**—as well as the execution traces of each experiment executed during the design process. The finite-state machine is then modified by a designer—either a human or an automatic procedure—producing a modified finite-state machine—such as the one produced by removing a state shown in panel **(D)** where the state and the relative transitions that have been removed are shown in light gray—or by modifying the parameters. First, the state values of the original finite-state machine are calculated from the execution logs using the *first-visit* MC method. Importance sampling uses the state values, the modified finite-state machine, and the execution traces to calculate the estimated state values of the modified finite-state machine that are then used to produce an estimation of the performance of the modified finite-state machine. Finally, in panel **(B),** we show a screenshot from the ARGoS simulator where a swarm of 20 robots is performing the foraging task using the control software shown in panel **(C)**.

In AutoMoDe, the control software is generated by combining pre-existing parametric software modules in a modular architecture, such as a probabilistic finite-state machine or a behavior tree. When considering finite-state machines, the software modules are either state modules and transition modules. The optimization algorithm designs control software by optimizing the structure of the PFSM—the number of states and how they are connected to each other—the behaviors, the transitions, and their parameters. In Figure 1C, we show an example of a finite-state machine generated using AutoMoDe.

Regardless of the design method and control software architecture, once generated, the control software is improved through an iterative process where changes are evaluated and applied if considered promising. For instance, a human designer applies this process when fine-tuning the configuration of the control software. The optimization algorithms used in automatic design methods, and in particular iterative optimization methods, also work in this way. For instance, iterative optimization methods start from one or multiple solutions and explore the solution space in an iterative fashion. At each iteration, the optimization process generates new solutions by considering modifications of the previous solution(s). Having an estimate of the performance of such modifications can save valuable resources—access to appropriate computational resources can be expensive—and significantly speed up the design process. Furthermore, in the context of automatic design, such an estimate could be used to reduce the complexity of the generated control software. Indeed, the automatic design process often introduces artifacts in the generated control software, that is, there may be parts of the control software that do not contribute to the performance because they either do not influence the behavior of the robots or are never executed. These artifacts are generally ignored, but they add unnecessary complexity and hinder readability.

Our proposal is to use off-policy evaluation to estimate the impact of a modification from data collected during the execution of the control software. Off-policy evaluation, is a technique developed in the context of reinforcement learning (Bertsekas and Tsitsiklis, 1996; Sutton and Barto, 2018), to estimate the performance of a *target* policy from the observation of the one of a *behavior* policy. In a policy, the world is represented as a set of states—possible configurations of the environment, including the robot itself—connected by actions—the possible interactions with the world. Each state is associated with a set of possible actions that can be taken to transition from one state to the other. The target policy may be deterministic—given a state it always executes the same action—or stochastic—the action to be performed is chosen with a certain probability; while the behavior policy must be stochastic (Sutton and Barto, 2018).

Almost all off-policy methods use a technique called importance sampling (Hammersley and Handscomb, 1964; Rubinstein and Kroese, 1981; Sutton and Barto, 2018). This technique is used to compute a statistic of a distribution based on a sample extracted from another. In this way, data generated by one policy can be used—after being appropriately weighted through importance sampling—to evaluate a different policy. For instance, one may execute a policy that performs actions randomly and use the observed policy performance to estimate the performance of a deterministic policy that always chooses the action with the highest expected reward. Given a set of actions executed by the behavior policy, this technique estimates the performance of the target policy from the one observed by performing the behavior policy by weighting the latter with the ratio between the probability of executing each action under the target policy and the one of executing them under the behavior policy. As a consequence, the behavior policy must contain all the states and actions of the target policy. Moreover, under the behavior policy, the probabilities of executing each action under each state must be strictly positive.

Off-policy methods based on importance sampling have been studied in reinforcement learning for a long time (Hammersley and Handscomb, 1964; Powell and Swann, 1966; Rubinstein and Kroese, 1981; Sutton and Barto, 2018). Recent works focused on combining importance sampling with temporal difference learning and approximation methods (Precup et al., 2000, Precup et al., 2001), as well as reducing the variance of the estimation (Jiang and Li, 2016) and improving the bias-variance trade-off (Thomas and Brunskill, 2016).

The control software of a robot is indeed the implementation of a policy. In fact, a robot uses information acquired through its sensors to acquire information on the world and execute an action by properly operating its actuators and motors. Depending on the control software architecture, the state might be explicitly reconstructed or not, and the set of actions available in each state might be defined in a more or less explicit way. In the case of probabilistic finite-state machines, the similarities of this control software architecture with policies makes it ideal for this study. By considering additional information from the sensors of the robot, the states and transitions of a PFSM are directly translated in states and actions of a policy. In the technique we propose in this paper, the relevant data is the execution traces—that is, the sequence of internal states traversed by the controller and the sequence of sensor readings—from each robot in the swarm during multiple experimental runs.

To evaluate the technique we propose, we use control software generated with a variant of Chocolate (Francesca et al., 2015) that we modified to allow the generation of more complex finite-state machines composed of up to six states. In order to avoid confusion, we call this variant Chocolate−6S . A further advantage of using AutoMoDe is that collecting the execution data needed for the estimation does not require additional experimental runs because the technique we propose can operate on the data produced within the design process. In the experiments, we consider two kind of modifications, one concerning the structure and one the parameters of the control software. In the first, we estimate the performance after removing one of the states of the control software. In the second, we evaluate the impact of modifying the values of two parameters of the two most executed transitions. In both experiments, we compared the estimation provided by the proposed technique with a naive estimation made assuming that the applied modification would not change the performance. The results show that the proposed technique is better than the naive estimation and that the difference between the two is statistically significant.

The structure of the paper is the following. In Section 2, we present off-policy evaluation and how it can be applied to finite-state machines. The experiments, their setup, and results are presented in Section 3. Finally in Section 4, we discuss the limitations of the proposed technique, possible ways to improve the estimation and how it can be extended to other control software architectures.

## 2 Method

### 2.1 Background: Off-Policy Evaluation

The main challenge in reinforcement learning is estimating how desirable it is for an agent to be in a state. The value $v_{\pi }\left(s\right)$ of a state *s* under a policy π is the expected reward obtainable by starting in state *s* and then following the policy π. The reward is calculated over an episode *e* that can be defined as an entire interaction between an agent and the environment, from an initial to a final step. The reward obtained after step *t* can be calculated as

$$G_{t}\left(s;e\right)≐R_{t+1}+\gamma G_{t+1}.$$ (1)

In Eq. 1, $R_{t+1}$ is the reward obtained at step t+1 and γ∈[0,1] is a parameter called discount rate, which allows us to model the fact that rewards may depend less and less on states visited earlier in the episode.

Using Monte Carlo (MC) methods, the value function can be estimated from a sample of episodes. In these methods, the value $v_{\pi }\left(s\right)$ of a state *s* is calculated as the average of the returns following the visit to state *s*. In the *first-visit* MC method only the reward following the first visit is considered while in *every-visit* all the visits contribute to the average. As the two methods are similar and both converge to $v_{\pi }\left(s\right)$ when the number of visits tends to infinity, to simplify calculations in this work we use the *first-visit* MC method.

Off-policy evaluation estimates the value function of a policy π from episodes generated by another policy *b*. To be able to use episodes from *b* to estimate values for π, *b* must *cover* π, that is, it must be possible—under *b*—to take every action that can be taken under π. Formally, if π(a|s)>0 then b(a|s)>0, where π(a|s) indicates the probability under policy π of taking action *a* when in state *s*. Assuming that *b* covers π, importance sampling can be used to weight the returns of *b* considering that—between the policies—each action may be taken with a different probability. Defining τ(s) as the sequence of states and actions $\;S_{t},A_{t},S_{t+1},A_{t+1},\ldots ,S_{T}$—where $S_{t}$ is the first visit to state *s*, $A_{t}$ is the action taken when in $S_{t}$ and $S_{T}$ is the final state—the ratio between the different probabilities—called importance sampling ratio—can be expressed as

$$\rho_{\tau \left(s\right)}=\prod_{k=t}^{T-1}\frac{\pi \left(A_{k}|S_{k}\right)}{b\left(A_{k}|S_{k}\right)}.$$ (2)

In Eq. 2, $\pi \left(A_{k}|S_{k}\right)$ and $b\left(A_{k}|S_{k}\right)$ indicate the probability of taking action $A_{k}$ when in state $S_{k}$ under the target policy and the behavior policy, respectively. Given a set of episodes *E* generated with policy *b*, there are two main ways of using $\rho_{\tau \left(s\right)}$ to estimate $v_{\pi }\left(s\right)$: *ordinary importance sampling* and *weighted importance sampling* (WIS). *Ordinary importance sampling* is defined as

$$v_{\pi }\left(s\right)=\frac{\sum_{e\in E}\rho_{\tau \left(s\right)}\left(e\right)G_{t}\left(s;e\right)}{\left|E\right|}.$$ (3)

Weighted importance sampling instead is defined as

$$v_{\pi }\left(s\right)=\frac{\sum_{e\in E}\rho_{\tau \left(s\right)}\left(e\right)G_{t}\left(s;e\right)}{\sum_{e\in E}\rho_{\tau \left(s\right)}\left(e\right)}.$$ (4)

The main difference between Eqs. 3, 4 is that, in the latter equation, $\rho_{\tau \left(s\right)}$ is in the denominator. Both *ordinary importance sampling* and *weighted importance sampling* tend to the exact state value when increasing the number of episodes considered but the two methods show a different variance and bias trade-off. *Ordinary importance sampling* is unbiased but shows a very high variance, *weighted importance sampling* introduces some bias resulting in a far lower variance (Sutton and Barto, 2018). We selected WIS for our implementation because during some preliminary experiments the variance of the estimation was found to impact significantly the results.

### 2.2 Our Technique: Applying Off-Policy Evaluation to Finite-State Machines

Applying off-policy evaluation to finite-state machines requires establishing what are states and actions in a PFSM, what is an episode and how to calculate the final reward, $G_{T}$, from the score attained by the whole swarm at the end of an experimental run. In finite-state machines, each state represents a behavior that is executed until an event triggers a transition to another state. Considering that the robot does not change its behavior until a transition is triggered, our working hypothesis is that a state of a PFSM–together with the information from the robot sensors saved in the execution traces—can be simplified in a single state of a policy and, consequently, transitions acquire the same function as actions in a policy. With these assumptions—considering a PFSM π—π(a|s) can be defined as the probability that transition *a* is triggered when in state *s*.

Naturally, an episode should correspond to an experimental run with the reward being the final score, but in swarm robotics there are several robots—typically all running the same control software. For this reason, we divide an experimental run involving a swarm of *n* robots in a set of *n* parallel episodes. Similarly, assuming *F* is the final score of the experimental run, the reward awarded to each robot corresponds to F/n. In calculating $G_{T}$ using Eq. 1, because assigning a per-time-step reward is not always possible, we set $R_{t+1}=0$—that is, the reward is given only at the end of the episode—and we do not consider any discount—that is, γ=1—resulting in $G_{T}=F/n$. Using the *first-visit* method, a state will get a reward of $G_{T}$ if it is executed at least once during the episode. In this case, a state that is executed once—for instance, the initial state—will have the same reward as a state that is executed for almost the entirety of the episode. We also considered another way of calculating $G_{T}$ that consists in weighting the reward by the relative execution time of each state. This proportional $G_{T}$ is calculated per state so that a state *s* that has been executed for *k* time steps gets a reward $GP_{T}\left(s\right)=\left(F/n\right)\cdot \left(k/\text{steps}\right)$ where steps is the total number of time steps in the episode. The pseudo code showing how to estimate the state values is shown in Algorithm 1.

Algorithm 1 Pseudo code showing how the state values are calculated. The inputs are the finite-state machine and its execution traces generated during the design process. Each execution trace contains the recording of all the robots in the swarm as well as the final score awarded to the swarm at the end of the experiment. The procedure iterates over each execution trace and for each execution trace considers each robot separately. For each robot, the value of each state is calculated using the *first-visit* MC method and considering two ways of calculating the reward. In the first, the reward is equal for each state and is equal to the reward per robot. In the second, each state has a reward calculated as a fraction of the reward per robot proportional to the total time the state was active.

 **Input** A PFSM composed of *S* states and *T* transitions.
 **Input** A set of execution traces
 **Output** V(s) state values using the full reward
 **Output** $V_{p}\left(s\right)$ state values using the proportional reward
 **for** each s∈S_**do**_
** ** **Initialize** Rewards(s) as an empty list
** ** **Initialize** ${\text{Rewards}}_{p}\left(s\right)$ as an empty list
 **loop** over each execution trace
** ** F= final score of the swarm
** ** n=number of robots in the swarm
** ** $G_{T}=F/n$ reward per robot
** ** steps= number of time steps in the execution trace
** ** **for** each robot in the swarm **do**
**  ** **for** each state s∈S **do**
**   ** **if** *s* has been executed in the episode **then**
**    ** k= number of time steps *s* has been executed
**    ** Append $G_{T}$ to Rewards(s)
**    ** Append $G_{T}\cdot k/\text{steps}$ to ${\text{Rewards}}_{p}\left(s\right)$
 V(s)=average(Rewards(s))
 $V_{p}\left(s\right)=\text{average}\left({\text{Rewards}}_{p}\left(s\right)\right)$

Given these definitions, we can use *weighted importance sampling* to estimate the state values of a *target* finite-state machine from the execution traces of a *behavior* finite-state machine, provided that the states of the behavior finite-state machine are a superset of those of the target one and are connected by transitions in the same way. To calculate a performance estimation for the whole swarm when executing the target control software, F/n has to be derived from the state values estimated using *weighted importance sampling*. Let $v_{b}\left(s\right)$, with s∈S be the state values of the behavior finite-state machine and $v_{t}\left(j\right)$, with j∈J and J⊆S, be the state values of the target finite-state machine estimated using *weighted importance sampling*. When considering the reward calculated as $G_{T}=F/n$, the performance $F_{e}$ of the target finite-state machine is

$$F_{e}=\frac{\sum_{J}\left(v_{t}\left(j\right)/v_{b}\left(j\right)\right)}{\left|J\right|}\cdot G_{T}.$$ (5)

In Eq. 5, the estimation is calculated as the per-robot reward multiplied by a factor that combines the estimated state values of the target finite-state machine weighted by the state values of the behavior finite-state machine. The proportional reward already considers the relative contribution of each state so the calculation of $F_{e}$ is defined as follows:

$$F_{e}=\sum_{J}v_{t}\left(j\right)GP_{T}.$$ (6)

$F_{e}$ represents the estimation of the average performance of a single robot in the swarm. The estimated performance of the swarm, considering the assumption that all the robots contribute equally, is calculate as $F_{e}\cdot n$.

The experiments presented in this paper are based on finite-state machines generated with Chocolate−6S, which builds PFSM composed of a maximum of six states and four transitions per state. Each state can assume one of six behaviors and each transition can have one of six conditions. The key characteristics of Chocolate−6S, as well as a brief description of the behaviors and the conditions, are given in Table 1. To produce execution traces for each experimental run, we modified AutoMoDe so that the control software of each robot logs an execution trace containing, for each time step, the current state, the active transition(s) and the information needed to calculate the activation probability of each condition—that is, the ground color and the number of neighboring robots perceived.

**TABLE 1:** Description of Chocolate−6S. This method targets the e-puck robot and specifically the reference model RM1.1 of which we report the key features at the end of the table. The finite-state machines are generated by choosing from six behaviors—for which we provide a brief description—and six conditions—for which we report how the activation probability *p* is calculated with α and β being parameters. Chocolate−6S can generate finite-state machines of maximum six states, while Chocolate allows a maximum of four and each state can have a maximum of four transitions. The optimization algorithm is iterated F-Race which uses the ARGoS simulator to perform evaluations.

Chocolate−6S		
**Modules**		
Behaviors	Exploration	Move randomly
	Stop	Stop moving
	Phototaxis	Move toward the light
	Anti-phototaxis	Move away from the light
	Attraction	Move toward other robots
	Repulsion	Move away from other robots
Conditions	Black-floor	If the floor is black, P=α; 0 otherwise
	White-floor	If the floor is white, P=α; 0 otherwise
	Gray-floor	If the floor is gray, P=α; 0 otherwise
	Neighbor-count	With *n* neighbors $P=1/\left(1+e^{\beta \cdot \left(\alpha -n\right)}\right)$
	Inverted-neighbor-count	With *n* neighbors $P=1-1/\left(1+e^{\beta \cdot \left(\alpha -n\right)}\right)$
	Fixed-probability	P=α
**Constraints**		
Number of states Transitions per state	6 (max)	
	4 (max)	
**Tools**		
Optimization algorithm Simulator	iterated F-Race implemented in the irace package (Balaprakash et al. (2007); López-Ibáñez et al. (2016))	
	ARGoS3 (Pinciroli et al. (2012))
**Robot platform**	e-puck reference model RM1.1 (Hasselmann et al. (2020))	
Input	8 proximity sensors	
	8 light sensors	
	3 ground sensors	
	Number of neighboring robots perceived	
	Attraction vector for each perceived robot	
Output	Left and right wheel target linear velocity	

## 3 Results

In the experiments presented here, the execution traces are collected during the generation of the control software from the executions performed by the optimization algorithm used in Chocolate−6S , Iterated F-Race implemented in the irace package (Birattari, 2009; López-Ibáez et al., 2016). In a nutshell, I/F-race works in an iterated fashion by generating, testing and discarding solutions—i.e., finite-state machines. In each iteration, I/F-race keeps a set of solutions that are executed and compared with each other. A solution is discarded when proved worse than the others by means of a statistical test. The algorithm ends when the maximum amount of executions is reached, returning the set of surviving solutions. Figure 1A shows a description of how a finite-state machine is generated and modified as well as how the performance of the modified control software is estimated.

We applied off-policy evaluation to 20 finite-state machines generated with Chocolate−6S to perform a foraging mission as defined by Francesca et al. (2014). In this mission, a swarm of 20 robots, confined in an dodecagonal arena, must retrieve as many objects as possible from two sources and transport them to the nest. A screenshot of an experimental run is shown in Figure 1B. The e-puck robot is not able to manipulate objects so the interaction with objects is abstracted. We consider that a robot collects an object by entering a source and deposits it by entering the nest. The sources are two black circles roughly in the middle of the arena while the nest is a white area placed at the edge of the arena. Additionally, a light source is placed behind the nest. The performance metric is defined as the number of objects retrieved. A video of an experimental run showcasing the mission as well as the source code and the experimental data are available in the supplementary page (Supplementary Material).

All the experiments were conducted in a simulated environment using ARGoS3 Pinciroli et al. (2012). We executed Chocolate−6S  ten times with a budget of 10,000 evaluations, generating 93 finite-state machines. From this group, we removed the finite-state machines that had less than three active states according to the execution traces. From the remaining ones, we formed the final group of 20 finite-state machines by selecting the ones showing the greatest number of active states. During the execution of Chocolate−6S, these finite-state machines were executed between seven and ten times. Considering that each experimental run involves twenty robots, we collected from 140 to 200 episodes for each finite-state machine.

We devised two experiments: one, called “state pruning,” in which we consider changes to the structure of the finite-state machines and one, called “parameter variation,” where we consider variations in its configuration. In state pruning, for each finite-state machine, we use the proposed technique to estimate the performance of all the finite-state machines that can be generated by removing one state—for instance, three finite-state machines composed of two states can be generated from one composed of three states. Figure 1D shows a finite-state machine composed of three states generated by removing state S2 from the finite-state machine in Figure 1C. In parameter variation, for each finite-state machine, we generate four variations by changing two parameters of the two most active transitions. For each parameter, we considered two different values generating four combinations per finite-state machine. For instance, we can generate four finite-state machines by considering two new values for the α parameters of the two transitions connecting the states S0 and S3 in the finite-state machine in Figure 1C. From these 80 combinations, we discarded the ones unable to change significantly the performance of the control software after five experimental runs, resulting in a total of 20 parameter variations. In both experiments, we compared the performance estimations calculated with weighted importance sampling—both with and without the proportional reward—with a naive estimation implemented as the average performance of the unmodified finite-state machine as reported in the execution traces. In other words, the naive estimation always assumes that the changes done to the control software will not influence its performance. We measured the accuracy of each estimation using the normalized squared error (SE):

$$\text{normalized SE}=\frac{{\left(\pi_{i}\left(E\right)-P_{i,b}\left(E\right)\right)}^{2}}{\pi_{i}\left(E\right)}.$$ (7)

In the equation, $\pi_{i}\left(E\right)$ is the measured average performance of the finite-state machine *i* over the set of executions *E* and $P_{i,b}\left(E\right)$ is the estimated performance calculated from the traces generated by the finite-state machine *b* within the set of executions *E*. Moreover, we tested the results for significance using the Friedman rank sum test.

**FIGURE 2:**
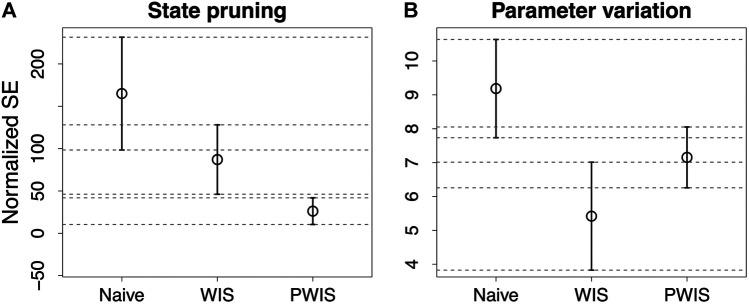
Results of the experiments measured as normalized mean squared error. In **(A)**, we report the results of estimating the impact of removing a state; in **(B)**, we report those of estimating the impact of changing the probabilities of the two most active transitions. Naive indicates the naive estimation, while WIS and PWIS indicate the results obtained with the weighted importance sampling using, respectively, the full and the proportional per state reward.

The results for the two experiments are shown in Figure 2 where, we indicate with WIS the result obtained using weighted importance sampling and with PWIS the result obtained using weighted importance sampling and the proportional reward. In both cases, PWIS and WIS have better results than the naive estimation with PWIS having significantly better results in the state pruning experiment—shown in Figure 2A—and WIS being significantly better in the parameter variation experiment—shown in Figure 2B. When comparing the results, the larger estimation error shown by all methods in the state pruning experiment can be explained by the fact that, in this experiment, the finite-state machines undergo substantial modifications which may invalidate the execution traces leading to performance estimation of 0. This is the case, for instance, when removing a state generates a finite-state machine with no transitions.

Overall, the results indicate that PWIS gives a better estimation when changing the structure of a finite-state machine while WIS is better suited to estimate the effect of variations to the parameters. In the first experiment, the proportional reward used in PWIS—that includes a measure of the relative execution time of each state—makes it better suited to estimate how the performance would change when removing a state. On the contrary, in the parameter variation experiment, the changes to the parameters influence directly the execution time of the states, making PWIS less accurate than WIS.

## 4 Discussion

In this paper, we applied off-policy evaluation to estimate the performance of a robot swarm where the control software is represented as a finite-state machine. Although the experiments deliver promising results, further experimentation is needed, considering different missions as well as different sets of software modules. However, the results indicate that this line of research is promising with several developments that could be explored such as different reward calculations, different estimators. The execution traces can be modified to also trace the performance metric of the swarm so that more complex reward calculations can be implemented. The estimation can be improved by employing importance sampling methods such as the ones proposed by Jiang and Li (2016); Thomas and Brunskill (2016).

Moreover, this technique is not necessarily limited to finite-state machines and it could be extended with some modifications to other modular control software architecture such as, for instance, behavior trees (Kuckling et al., 2018). Another interesting application of this technique would be in automatic design methods using iterative optimization algorithms. The execution time of these methods might be reduced by running simulations only if newly generated solutions have an estimated performance that is better than the current best one.

## Data Availability

The datasets presented in this study can be found in online repositories. The names of the repository/repositories and accession number (s) can be found as follows: http://iridia.ulb.ac.be/supp/IridiaSupp2020-012/index.html.
